# LncNetP, a systematical lncRNA prioritization approach based on ceRNA and disease phenotype association assumptions

**DOI:** 10.18632/oncotarget.23059

**Published:** 2017-12-08

**Authors:** Chaohan Xu, Yanyan Ping, Hongying Zhao, Shangwei Ning, Peng Xia, Weida Wang, Linyun Wan, Jie Li, Li Zhang, Lei Yu, Yun Xiao

**Affiliations:** ^1^ College of Bioinformatics Science and Technology, Harbin Medical University, Harbin, China; ^2^ Key Laboratory of Cardiovascular Medicine Research, Harbin Medical University, Ministry of Education, Harbin, China

**Keywords:** lncRNA prioritization, ceRNA theory, disease phenotype association, pan cancer

## Abstract

Our knowledge of lncRNA is very limited and discovering novel disease-related long non-coding RNA (lncRNA) has been a major research challenge in cancer studies. In this work, we developed an LncRNA Network-based Prioritization approach, named “LncNetP” based on the competing endogenous RNA (ceRNA) and disease phenotype association assumptions. Through application to 11 cancer types with 3089 common lncRNA and miRNA samples from the Cancer Genome Atlas (TCGA), our approach yielded an average area under the ROC curve (AUC) of 83.87%, with the highest AUC (95.22%) for renal cell carcinoma, by the leave-one-out cross validation strategy. Moreover, we demonstrated the excellent performance of our approach by evaluating the influencing factors including disease phenotype associations, known disease lncRNAs and the numbers of cancer types. Comparisons with previous methods further suggested the integrative importance of our approach. Taking hepatocellular carcinoma (LIHC) as a case study, we predicted four candidate lncRNA genes, RHPN1-AS1, AC007389.1, LINC01116 and BMS1P20 that may serve as novel disease risk factors for disease diagnosis and prognosis. In summary, our lncRNA prioritization strategy can efficiently identify disease-related lncRNAs and help researchers better understand the important roles of lncRNAs in human cancers.

## INTRODUCTION

At least 90 % of the human genome is actively transcribed, while protein-coding gene only accounts for ∼2% of the genome sequences. The rest of transcripts are non-coding RNAs including microRNAs (miRNAs) and long non-coding RNAs (lncRNAs) [1–4]. Within them, miRNAs have been identified to play important roles in cancer initiation, progression and metastasis, some of which may serve as potential biomarkers for cancer diagnosis and prognosis [4]. Compared to miRNAs, lncRNAs, a class of non-protein coding transcripts that are longer than 200 nucleotides without protein-coding capacity, have been also identified to regulate key cellular processes in carcinogenesis [1–3]. Currently, more than 12000 lncRNAs encoded in the human genome have been identified. Systematical studies revealed some “oncogenes” and “tumor suppressors” lncRNAs in cancer [5]. Despite much progress made by high-throughput biological techniques, the identification of cancer-related lncRNAs has remained a great challenge for researchers.

Towards this, several computational approaches have been developed to prioritize candidate disease lncRNAs and aim to improve the prediction performance in lncRNA prioritization [6–18]. For example, Yang *et al*. presented a propagation algorithm to uncover lncRNAs and disease associations through construction of a coding-non-coding gene disease bipartite network. They applied the lncRNA prioritization approach to 103 diseases and achieved an Area under ROC curve (AUC) of 0.7881 by leave-one-out cross validation [6]. In another study, Chen *et al*. presented a computational model, named LncRNA-Disease Association inference (HGLDA), to predict lncRNA-disease associations by integrating miRNA-disease associations and lncRNA-miRNA interactions and this approach obtained an AUC of 0.7621 in the leave-one-out cross validation [7]. Considering the important roles of lncRNAs in complex diseases, prioritization of candidate disease lncRNAs could not only benefit the understanding the underlying disease mechanism at the lncRNA level, but also facilitate the identification of disease biomarkers for disease diagnosis, treatment and prognosis. Moreover, several studies have demonstrated that lncRNA-related competing endogenous RNA (ceRNA) patterns have been widely found in human diseases, especially in cancers [7, 8]. Based on biological experiments or RNA sequencing techniques, some studies have been proposed to identify potential lncRNA-related ceRNA interactions and further investigate their functions. Li and colleagues developed starBase v2.0 (http://starbase.sysu.edu.cn/) to systematically identify the ceRNA interaction networks from 108 CLIP-Seq (PAR-CLIP, HITS-CLIP, iCLIP, CLASH) data sets generated by 37 independent studies [19]. In another lncRNA-related ceRNA database, NPInter v3.0, interactions pertaining to ncRNAs were not only manually curated from scientific literature but also curated from high-throughput technologies. Additionally, lncRNA-miRNA interactions from *in silico* predictions supported by AGO CLIP-seq data were also collected to estimate their potential ceRNA relationship [20]. Such ceRNA hypothesis-based studies provided valuable resources of relationships between lncRNAs with protein-coding genes, which can help infer lncRNA-disease associations in diverse human diseases by means of known disease knowledge.

Moreover, disease phenotype associations (namely disease phenotype similarities) enable improving the limitation that lacks sufficient disease knowledge, which have been successfully applied in prioritization of candidate disease miRNAs or genes [21, 22]. Recently, disease phenotype association-based computational approaches for prioritizing disease-specific non-coding RNAs have also emerged [9–13, 21, 23]. Such studies suggested that diseases with phenotypic similarity tend to show more close relations and their relevant RNA molecules often interact with each other in the interaction networks or form the similar physical or functional modules. Therefore, combination of knowledge from multiple diseases can provide additional clues for a specific disease. Even for those diseases without any known disease information, other associated diseases can be used to capture their potential disease molecules.

**Figure 1:**
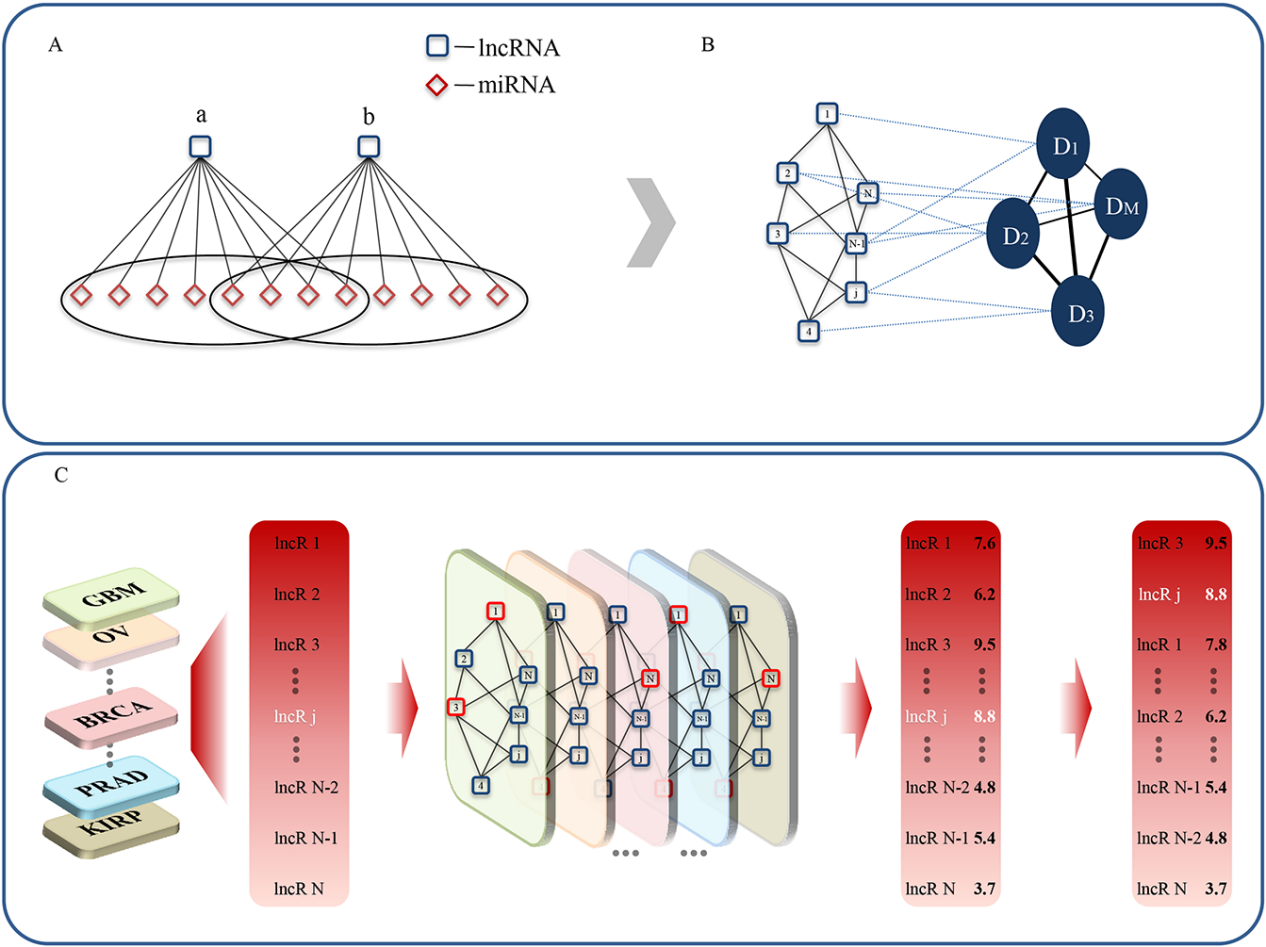
The workflow of LncNetP (**A**) Identification of significant lncRNA-lncRNA interactions according to miRNAs with ceRNA relations. (**B**) Construction of cancer-specific lncRNA networks associated with different disease phenotypes. (**C**) Candidate disease lncRNA prioritization by integration of disease phenotype associations.

Therefore, we combined the ceRNA theory and the disease phenotype association assumption to propose a systematical lncRNA prioritization approach “LncNetP”. Through interrogation of RNA-seq datasets from TCGA (https://cancergenome.nih.gov/), we constructed lncRNA interaction networks for 11 cancer types. Utilizing known disease lncRNAs as seeds, we used random walk with restart (RWR) approach to prioritize candidate disease lncRNAs for each cancer type and integration of all prioritization results by the disease phenotype associations (Figure 1). The average AUC score of prioritization results across 11 cancer types is 83.87%, with the highest AUC being 95.22% for renal cell carcinoma. Our results show that through the integration of disease phenotype associations, the lncRNA prioritization performance can be improved, especially for some diseases with few or without known disease lncRNAs.

## RESULTS

### Systematic identification of lncRNA associations using the ceRNA assumption

For 11 cancer types, we obtained matched miRNA and lncRNA sequencing data that detected by IlluminaHiSeq miRNASeq and IlluminaHiSeq RNASeqV2 platforms, respectively, from TCGA database, including Bladder urothelial carcinoma (BLCA), Breast invasive carcinoma (BRCA), Cervical squamous cell carcinoma and endocervical adenocarcinoma (CESC), Kidney renal clear cell carcinoma (KIRC), Brain lower grade glioma (LGG), Liver hepatocellular carcinoma (LIHC), Lung adenocarcinoma (LUAD), Prostate adenocarcinoma (PRAD), Stomach adenocarcinoma (STAD), Thyroid carcinoma (THCA) and Uterine corpus endometrioid carcinoma (UCEC). A total of 3089 disease samples were contained (Supplementary Table 1).

Through mapping miRNAs and lncRNAs to GENCODE [2] and miRBase [24] databases, 1034 mature miRNAs and 12727 lncRNAs were obtained. Subsequently, we calculated miRNA-lncRNA co-expression relations by Pearson correlation coefficient (PCC) and retained the significant miRNA-lncRNA relationships with false discovery rate (FDR) less than 0.05 (Benjamini and Hochberg correction). To further increase the credibility between lncRNAs and miRNAs, we combined lncRNA and miRNA interaction pairs derived from starBaseV2.0 [19] and NPInter [20] databases, which involved 10169 lncRNA-miRNA interactions. To further check the ratios of lncRNA-miRNA pairs having the same biological functions, we carried out the enrichment analysis between GO gene sets from MSigDB (http://software.broadinstitute.org/gsea/msigdb/) and lncRNAs’ co-expressed genes and miRNA target genes from miRTarBase (http://mirtarbase.mbc.nctu.edu.tw/php/index.php) by a hypergeometric test. Then, miRNA and lncRNA pairs with significant relationships (Benjamini-Hochberg correction, *p* ≤ 0.05) for each GO term were recorded as pairs with the same biological functions. For the top 10% of lncRNAs in the candidate lncRNA lists of hepatocellular carcinoma (LIHC), breast cancer (BRCA) and prostate cancer (PRAD), we found 39.11% (24371 out of 62319), 41.76% (26368 out of 63118) and 45.37% (28656 out of 63167) miRNA-lncRNA pairs with the same biological functions, respectively (Benjamini-Hochberg correction, *p* ≤ 0.05, Supplementary Table 2). The results further supported the fundamental assumption of our proposed method.

Finally, a hypergeometric test was performed to excavate significant lncRNA-lncRNA competing pairs based on the hypothesis that lncRNA pairs share the same miRNA response elements (MREs), which would have the same or similar biological functions.

### Prioritization of candidate disease lncRNAs through combining disease-specific lncRNA networks and disease phenotype associations

Based on the ‘guilt-by-association’ hypothesis that disease-related lncRNAs with similar expression have the same or similar functions, we systematically prioritized candidate disease lncRNAs based on the disease-specific lncRNA networks. Hence, a disease-specific lncRNA network for a given cancer type was constructed according to the lncRNA-lncRNA relations. Each edge in the lncRNA network was weighted by the function of –log_10_^*P-value*^, in which *P*-*value* represents the significance of functional similarity between two lncRNAs in the specific cancer type. Subsequently, eleven lncRNA networks were constructed and relevant network topology characteristics were summarized in Supplementary Table 3.

The random walk with restart (RWR) approach was then used to prioritize candidate disease lncRNAs by the known disease lncRNAs from the Lnc2Cancer [25] database (http://www.bio-bigdata.net/lnc2cancer/) in each cancer type (see Methods). As a result, eleven candidate lncRNA lists representing the prioritization results of eleven cancer types were obtained. Based on the assumption that diverse diseases with phenotype associations show similar molecular mechanisms, we integrated all disease lncRNA prioritization lists by using the disease phenotype associations to quantify the links between candidate disease lncRNAs and cancers. We obtained all disease phenotype similarity scores from the ‘HPOSim’ package and extracted the phenotype association scores for the 11 cancer types. Finally, all candidate disease lncRNAs were ranked across their corresponding integrated prediction scores (For details, please see Figure 1).

### Evaluation of the performance by the leave-one-out-cross validation

**Figure 2:**
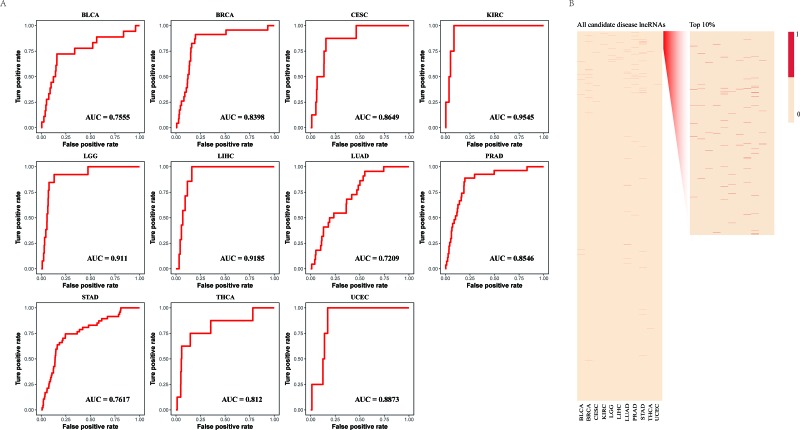
Evaluation of the performance of LncNetP (**A**) The ROC curves of lncRNA prioritization results. (**B**) Top 10% ranks of known disease lncRNAs after prioritization.

Furthermore, the leave-one-out cross-validation (LOOCV) strategy was then carried out to test the performance of our lncRNA prioritization approach based on experimentally verified disease-lncRNA associations from the Lnc2Cancer database. The average score of the area under the receiver operating characteristic (ROC) curve (AUC) yielded by our lncRNA prioritization approach was 83.87% (Figure 2A and 2B), strongly supporting that our approach has good prioritization performance in prioritization of candidate disease lncRNAs. Notably, 75 known disease lncRNAs in eleven cancer types were ranked in the top 30% in all candidate disease lncRNA lists. In particular, known disease lncRNAs, MEG3 and MALAT1, frequently occurred in 9 and 10 cancer types in the top 10% of candidate disease lncRNA lists, respectively.

### Evaluation of the robustness of lncRNA prioritization approach

We suspected that some factors, including disease phenotype associations, known disease lncRNAs and the number of disease studies, could influence the performance of our lncRNA prioritization approach. Therefore, it was necessary and important to evaluate their contributions to the performance in our lncRNA prioritization approach.

### Disease phenotype associations

**Figure 3:**
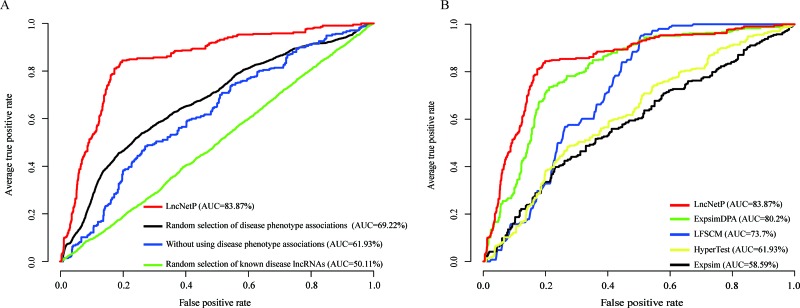
Evaluation of the robustness of LncNetP (**A**) Evaluation by randomly selecting disease phenotype associations with 1000 repetitions, excluding disease phenotype associations, and randomly selecting known disease lncRNAs with 1000 times. (**B**) The comparison results of LncNetP with HyperTest, LFSCM, Expsim and ExpsimDPA.

Eleven disease phenotype associations were used to characterize relationships between diseases and provide the promise to elucidate the pathogenesis mechanisms of diseases in the crosstalk pattern. For evaluation of the importance of disease phenotype associations, we prioritized candidate disease lncRNAs in diverse cancer types without utilizing any disease phenotype associations. The average AUC based on known disease lncRNAs from Lnc2Cancer was 61.93%, lower than the AUC score (83.87%) with the inclusion of disease phenotype associations (Figure 3A). Notably, the AUC score for KIRC dropped from 95.2% to 64.4%, which suggested that the disease phenotype association enables to greatly complement the incomplete information of some diseases. In addition, we evaluated the efficiency of disease phenotype associations by random selections of disease phenotype associations with 1000 repetitions and we found the average AUC score (69.22%) of 1000 prioritization results was lower than the primary result of 83.87% (Figure 3A).

To further assess the prediction power of our approach, we performed the prioritization of candidate disease lncRNAs for each cancer type only dependent on disease lncRNAs of other cancers. Surprisingly, the yielded average AUC was 82.61%, supporting that our lncRNA prioritization approach has superior performance in predicting of potential risk lncRNAs (Supplementary Figure 1A), even for some diseases with little or without known disease information.

### The number of cancer types

Disease phenotype associations characterize the similarity of diseases in a cross-talk pattern and can efficiently assists to improve the performance of our lncRNA prioritization approach, especially for disease with few or without known disease lncRNAs. Hence, we sought to assess whether integration of more caner types can improve the performance of our lncRNA prioritization approach. Towards this, we randomly selected 3, 5, 7 and 9 cancer types from the original eleven cancer types and re-computed prioritization scores for candidate disease lncRNAs. We found that upon increasing the number of cancer types for analysis, the average AUC scores were increased from 69.45% to 81.64% (Supplementary Figure 1B). Together, utilization of more diseases with their phenotype associations can facilitate to improve the performance of lncRNA prioritization.

### The number of known disease lncRNAs

Intuitively, our lncRNA prioritization approach may rely upon the number of known disease lncRNAs. Thus, it is necessary to evaluate the influence of known disease lncRNAs to our approach. Through random selections of the same number of non-disease associated lncRNAs as causal lncRNAs for each cancer type, new predication scores for all candidate disease lncRNAs of eleven cancer types were re-calculated and evaluated by LOOCV as described above. Owing to the lack of the non-disease lncRNA set, we generated a non-disease lncRNA set containing 12196 lncRNAs based on known disease lncRNAs from the Lnc2Cancer database. Equal numbers of non-disease lncRNAs for each cancer type were randomly selected 1000 times and used for prioritization. We obtained an average AUC score of 50.11% that was significantly lower than the primary prioritization results based on known disease lncRNAs (Figure 3A).

### Comparison with other lncRNA approaches

In addition, we compared our approach LncNetP with other lncRNA prioritization methods, which utilized the assumption that disease-related lncRNAs tend to show high functional associations. For example, the expression similarity (ExpSim) algorithm is based on a computational framework to accomplish lncRNA prioritization by combining human lncRNA expression profiles, gene expression profiles, and human disease-associated gene data [13]. In another study, the hypergeometric test (HyperTest) algorithm was proposed to infer disease lncRNA and disease-miRNA associations by evaluating the significance of common targets [7, 10]. Furthermore, the model of LncRNA Functional Similarity Calculation based on the information of MiRNA (LFSCM) was developed to calculate lncRNA functional similarity by combining disease semantic similarity, miRNA-disease associations and lncRNA-miRNA interactions [7]. We compared our lncRNA prioritization approach with these lncRNA prioritization approaches including HyperTest, LFSCM, ExpSim and improved ExpSim (named ExpSimDPA, which additionally integrated disease phenotype associations). The LOOCV analysis was then performed and AUC values generated by the above four lncRNA prioritization approaches were 61.93%, 73.7%, 58.59% and 80.2%, respectively. As a comparison, LncNetP has the highest AUC value (83.87%) in the LOOCV analysis (Figure 3B). The analysis results showed the outstanding performance of LncNetP.

### Case studies

**Figure 4:**
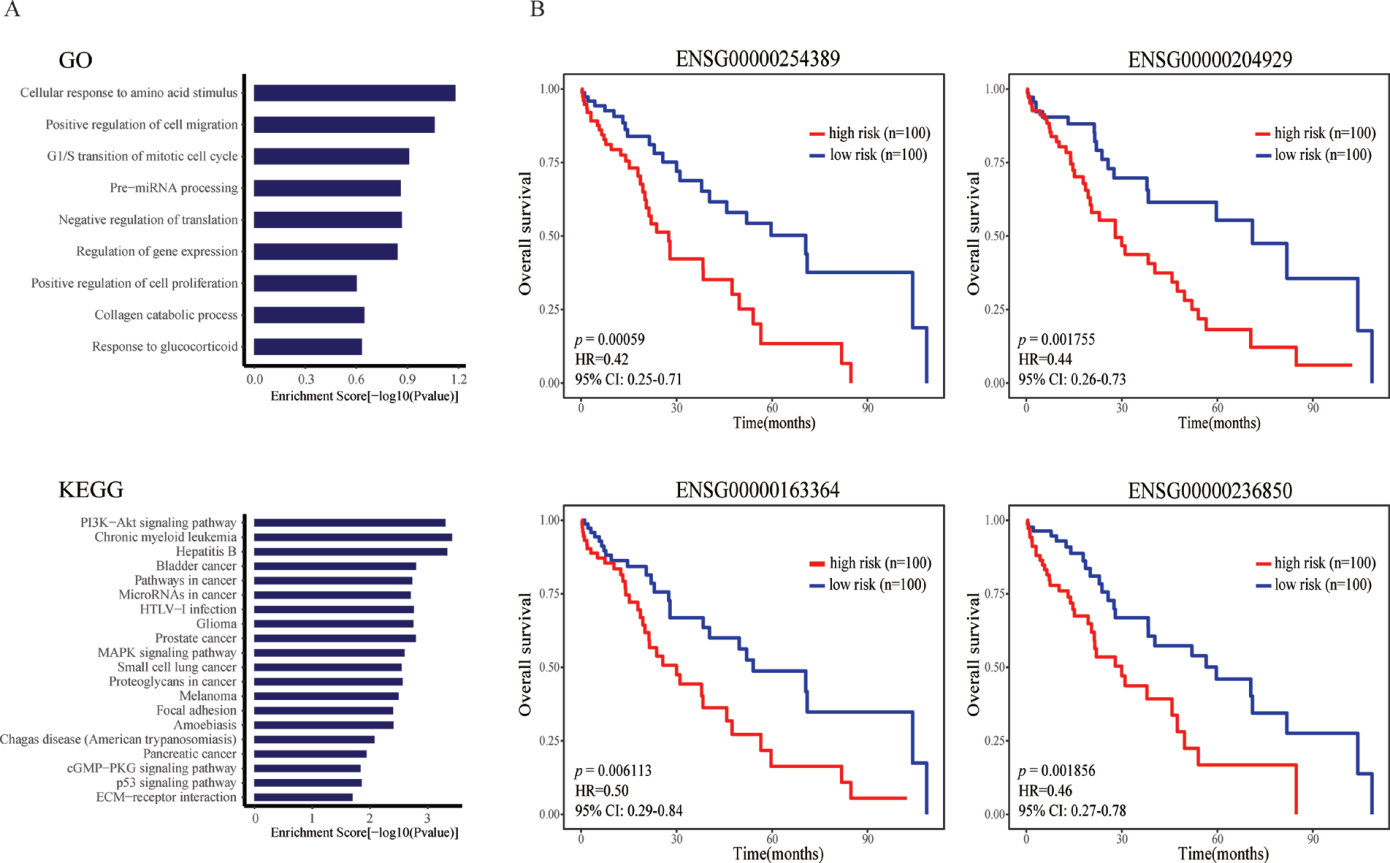
The prioritization results in the case study of LIHC (**A**) The GO and KEGG enrichment analysis results for top 10% lncRNAs of LIHC. (**B**) Survival analysis results of four candidate lncRNAs.

Liver hepatocellular carcinoma (LIHC) is a highly aggressive cancer, with the third leading cause of cancer mortality worldwide [26]. Taking LIHC as a case study, we used our lncRNA prioritization approach to identify and prioritize disease-related lncRNAs. Through evaluation by LOOCV, we found 6 out of 7 known disease lncRNAs, including ENSG00000130600 (H19), ENSG00000251562 (MALAT1), ENSG00000228630 (HOTAIR), ENS G00000251164 (HULC), ENSG00000099869 (IGF2-AS) and ENSG00000176840 (MIR7-3HG) ranked in the top 10 of the candidate list. To further validate the LIHC-related lncRNAs identified by our approach were high-confidence, we also chose the top 10% lncRNAs and investigated their potentially biological functions by function enrichment analysis of their associated genes as described in Method using the DAVID (https://david.ncifcrf.gov/, Benjamini test, *p* = 0.05) (Figure 4A). We found that these lncRNA-related genes were significantly enriched in LIHC-related GO terms and KEGG pathways. The GO terms include “G1/S transition of mitotic cell cycle”, “cellular response to amino acid stimulus”, “collagen catabolic process”, “cell division” and “negative regulation of translation”. while the KEGG enrichment analysis results contain “Chronic myeloid leukemia”, “Bladder cancer”, “PI3K−Akt signaling pathway”, “Pathways in cancer”, “MAPK signaling pathway”, “Proteoglycans in cancer”, “Cell cycle”, “p53 signaling pathway”.

In addition, we also predicted some candidate lncRNAs within them that have the most probability to be independent prognostic factors for LIHC survival. A univariate Cox proportional hazards regression analysis was performed to test whether the expression level of lncRNAs in the top 10% was significantly associated with survival of LIHC patients. Consequently, four survival-related lncRNAs including ENSG00 000254389 (RHPN1-AS1), ENSG00000204929 (AC0743 91.1), ENSG00000163364 (LINC01116) and ENSG000 00236850 (BMS1P20) were found and some of them were significantly correlated with the pathogenesis, development and metastasis of cancers (Figure 4B). RHPN1 antisense RNA 1 (RHPN1-AS1) knockdown significantly inhibited uveal melanoma (UM) cell proliferation and migration *in vitro* and *in vivo*. Liu *et al*. suggested that RHPN1-AS1 may serve as a candidate prognostic biomarker and target for new therapies in malignant UM [27]. LINC01116 was overexpressed in several cancers, and was transcriptionally repressed after Sulforaphane (SFN) treatment. The results from Beaver *et al*. supported an oncogenic function for LINC01116 in PC-3 cells when it was disrupted through the CRISPR/CAS9 method and confirmed knockdown of LINC01116 with siRNA decreased proliferation of prostate cancer cells [28]. In addition, Chung *et al*. found the overexpressed BMS1P20 may play potential functions in anaplastic large-cell lymphoma (ALCL) progression. They measured the expression levels of BMS1P20 in three cell lines by qRT-PCR and analyzed differences using a Kruskal-Wallis test (*P* < 0.05).

Besides of LIHC, additional two case studies, prostate cancer (PRAD) and breast cancer (BRCA), were also used to predict and identify disease-related lncRNAs. Twelve and six known disease related lncRNAs, such as ENSG00000251562 (MALAT1), ENSG00000130600 (H19), ENSG00000245532 (NEAT1), ENSG00000214548 (MEG3) and ENSG00000225937 (PCA3), were identified within the top 10% of the candidate disease lncRNA lists in PRAD and BRCA, respectively. Functional enrichment analysis showed that the top 10% of candidate lncRNAs for these two cancers were significantly enriched in many BRCA-related and PRAD-related GO functions and KEGG pathways (Supplementary Figures 2 and 3). Meanwhile, five survival-related lncRNAs in BRCA including ENSG00000238197 (PAXBP1-AS1), ENSG00000264515 (CTC-525D6.1), ENSG00000228327 (RP11-206L10.2), ENSG00000239407 (LL0XNC01-237H1.2) and ENSG00000238009 (RP11-34P13.7), and two survival-related lncRNAs in PRAD including ENSG00000272849 (RP11-347I19.8) and ENSG000 00238045 (AC009133.12), were found, with strong correlations with the metastasis of BRCA and PRAD (Supplementary Figures 2 and 3). Our findings suggested that these potential lncRNAs may promote the development of cancers and could serve as novel prognostic markers.

## DISCUSSION

Integration of different biological datasets for the accurate prediction of disease-related lncRNAs has become a critical challenge for understanding disease mechanisms. Fortunately, disease phenotype associations provide potential opportunities to supplement the incomplete information of known disease lncRNAs in a cross manner. The ceRNA relationships enable to create links of lncRNAs with a large number of protein-coding genes. Based on disease phenotype associations and ceRNA relationships, we therefore developed a computational pipeline through integration of large-scale RNA-seq datasets to systematically identify and prioritize candidate disease lncRNAs in the pan-cancer data set. And we found the combined application can indeed jointly improve the prediction power for prioritization.

According to the ceRNA hypothesis, high confidential lncRNA-miRNA relationships were obtained through the integration of co-expression relations from RNA-seq data from a specific cancer type and experientially verified relations from starBaseV2.0 and NPInter. Through the hypergeometric test, significant associations in each lncRNA pair according to their associated miRNA sets were generated, which ensures the rational construction of the disease-specific lncRNA network. Furthermore, a prediction score for each node in the lncRNA network assigned by the RWR approach based on known disease lncRNAs also efficiently guarantees the accuracy of identification of the associations between candidate lncRNAs and diseases. On the other hand, since the incomplete information of known disease lncRNAs exists in cancers, the assumption of disease phenotype associations therefore plays the crucial roles in integrating different disease phenotype so as to benefit the systematic identification of disease-related lncRNAs and facilitate in-depth understanding of their pathogenesis in human cancers. We manually checked the predicted lncRNA lists and found that several novel candidate disease lncRNAs in the top rank were newly verified by relevant databases or in recent experimental studies, which showed that they have high probabilities of being bona fide disease-related lncRNAs.

There are several limitations in our lncRNA prioritization approach. Firstly, relatively strict thresholds were set for the satisfaction of identifying high confidential lncRNA pairs with ceRNA relations. Some cancer types without matched lncRNA and miRNA expression data are not suitable for our lncRNA prioritization approach. Secondly, our lncRNA prioritization approach was restricted to disease phenotypes provided by‘HPOSim’ package. The disease phenotype similarity only characterizes the disease phenotype associations for human diseases without considering their corresponding subtypes. Finally, because of the limitation in evaluation of approach by LOOCV, some cancer types with only one to two known disease lncRNAs were not suitable for the lncRNA prioritization by our approach despite of the good prediction performance generated by our lncRNA prioritization.

In summary, we presented an integrated lncRNA prioritization approach for systematically prioritizing candidate disease lncRNAs associated with human disease. This approach can be used to facilitate the identification of disease-related lncRNAs and to increase the understanding of lncRNA-mediated pathogenesis. Using our approach, we performed overall prioritization of the candidate disease lncRNAs for eleven cancer types, which provided testable hypotheses to guide further experiments.

## MATERIALS AND METHODS

### LncRNA and miRNA sequencing data

All available cancer-related lncRNA and miRNA sequencing data, detected by miRNA-Seq (IlluminaHiSeq miRNASeq) and RNA-seq (IlluminaHiSeq RNASeqV2) sequencing platforms, were obtained from TANRIC (http://ibl.mdanderson.org/tanric/_design/basic/index.html) and FIREHOSE (http://gdac.broadinstitute.org/) (Supplementary Table 1). To comprehensively annotate human lncRNA and miRNA for further analysis, we collected 12727 lncRNAs and their corresponding annotation information from GENCODE, which combines the HAVANA manual annotation and Ensembl automatic annotation pipelines. Mature miRNA information was obtained from miRBase (release 21) that consists of 1034 mature miRNAs. All known disease lncRNAs were collected from Lnc2Cancer (http://www.bio-bigdata.net/lnc2cancer/).

### Identification of potential ceRNA interactions

To obtain the high confidential ceRNA relationships between lncRNAs and miRNAs, we extracted experimentally verified lncRNA-miRNA pairs from starBaseV2.0 and NPInter (http://www.bioinfo.org/NPInter/) [V3.0]. Totally, 10169 miRNA-lncRNA pairs containing 1663 lncRNAs and 246 miRNAs were obtained. To further identify lncRNA-miRNA pairs occurred in a specific cancer type, miRNA-lncRNA pairs with significant co-expression relationships (Pearson correlation coefficient analysis, FDR ≤ 0.05) were selected and used to the following analysis. For efficient identification of the significant interactions between lncRNA_a_ and lncRNA_b_, a hypergeometric test was carried out for each lncRNA interaction pair by:

$$P=1-\overset{x}{\underset{t=0}{\sum }}\frac{\left(\begin{array}{l} \begin{array}{l} \begin{array}{l} K\\ t \end{array} \end{array} \end{array}\right)\left(\begin{array}{l} N-K\\ M-t \end{array}\right)}{\left(\begin{array}{l} N\\ M \end{array}\right)}$$

N represents the total number of lncRNAs in the human genome. K and M represent the numbers of miRNAs respectively associated with lncRNA_a_ and lncRNA_b_. And x represents the common number of miRNAs shared the common MREs with the lncRNA_a_ and lncRNA_b_. A multiple test correction using the Benjamini–Hochberg procedure was then performed and used to confirm the potential lncRNA-lncRNA interaction relations.

### Construction of disease-specific lncRNA networks

After acquisition of disease-specific lncRNA-lncRNA relations in a specific cancer type, the corresponding lncRNA network was constructed. In the lncRNA network, nodes denoted lncRNAs and edges were weighted by $-\log_{10}^{P-value}$ to characterize relationships between two lncRNAs. Subsequently, cancer-specific lncRNA networks of eleven cancer types were generated and used to prioritize candidate disease lncRNAs (Figure 1).

### Prioritization of candidate disease lncRNAs through integration of disease phenotype associations

For each cancer type, we prioritized candidate disease lncRNAs s based on the corresponding cancer-specific lncRNA network and applied RWR propagation approach to calculate prediction scores for candidate disease lncRNAs.

Given a query cancer type *i* (Figure 1), taking the known disease lncRNAs of this cancer type as seed nodes, we utilized RWR approach to compute prediction scores for each node in the lncRNA network. Based on the assumption that diverse diseases with phenotype associations show similar molecular mechanisms, we further combined disease phenotype similarity scores with the prediction scores of lncRNAs into a unique prioritization score *S_ij_* by:

$$S_{ik}=\sum_{j=1}^{N}{\text{P}}_{ij}*S_{jk}$$

Where *P_ij_* represents the disease phenotype similarity score between cancer type *i* and *j*, and *S_jk_* represents the corresponding prediction score for candidate lncRNA *K* in cancer type *j* (Figure 1). Disease phenotype similarity scores were derived from the “HPOSim” package. After prioritization, candidate disease lncRNAs were ranked by the prediction scores.

### Evaluation of the robustness and the integration importance of our prioritization approach

We evaluated the performance of our prioritization approach by known disease lncRNAs using ROC curve analysis, and the leave-one-out cross-validation (LOOCV) was carried out to assess the prioritization performance. Known causal lncRNAs were extracted from the Lnc2Cancer database, which contains 1057 manually curated associations between 531 lncRNAs and 86 human cancers.

To evaluate the robustness and the integration importance of our lncRNA prioritization approach, we accepted the evaluation strategies by leaving out or permuting relevant influence factors, included disease phenotype associations, the number of cancer types and known disease lncRNAs, and interrogated the changes in the prioritization results. Finally, we assessed the prediction performance of our prioritization approach in identifying disease-related lncRNAs for each cancer type by only using other disease information.

### LncRNA functional enrichment analysis

Functional enrichment analysis for the associated genes of the top 10% of lncRNAs in three case studies was performed by using the DAVID (https://david.ncifcrf.gov/). We obtained the experimentally verified miRNA-lncRNA relationships from starBase and NPInter, and lncRNA-gene co-expression relationships (PCC ≥ 0.4) generated by TCGA data. To obtain more credible lncRNA-gene relationships, we integrated miRNA and gene regulations from the RegNetwork database [29] and retrieved lncRNA and gene pairs that were regulated by the same miRNA and had the co-expressed relations. These lncRNA-gene pairs were used for the lncRNA functional enrichment analysis.

The above processing was implemented using the R software.

## SUPPLEMENTARY TABLES AND FIGURES


