# Supplementary material for: LncNetP, a systematical lncRNA prioritization approach based on ceRNA and disease phenotype association assumptions
Source: Oncotarget. 2017 Dec 8;8(70):114603–12. doi: 10.18632/oncotarget.23059 (PMC5777717; doi:10.18632/oncotarget.23059)
Supplement: Supplementary file 1 [file oncotarget-08-114603-s001.pdf]

LncNetP, a systematical lncRNA prioritization approach based on ceRNA and disease phenotype association assumptions

SUPPLEMENTARY MATERIALS

Supplementary Table 1: Summary of lncRNA and miRNA datasets in TCGA

OMIM ID	Cancer types	Full names	Common samples
109800	BLCA	Bladder urothelial carcinoma	232
114480	BRCA	Breast invasive carcinoma	403
603956	CESC	Cervical squamous cell carcinoma and endocervical adenocarcinoma	193
144700	KIRC	Kidney renal clear cell carcinoma	121
137800	LGG	Brain lower grade glioma	482
114550	LIHC	Liver hepatocellular carcinoma	147
211980	LUAD	Lung adenocarcinoma	369
176807	PRAD	Prostate adenocarcinoma	318
613659	STAD	Stomach adenocarcinoma	210
188550	THCA	Thyroid carcinoma	433
608089	UCEC	Uterine corpus endometrioid carcinoma	181

Supplementary Table 2: The statistics of the ratios of lncRNA-miRNA pairs that having the same biological functions

Cancer types	lncRNA-miRNA pairs in the top 10% of lncRNA lists	No.of miRNA-lncRNA pairs with the same GO functions	Ratios
BRCA	63118 (1273 lncRNAs and 718 miRNAs)	26368	41.76%
LIHC	62319 (1273 lncRNAs and 679 miRNAs)	24371	39.11%
PRAD	63167 (1273 lncRNAs and 690 miRNAs)	28656	45.37%

Supplementary Table 3: Summary of disease-specific lncRNA networks of eleven cancer types

Cancer types	Nodes	Edges
BLCA	12680	33552608
BRCA	12683	31319979
CESC	12507	30419975
KIRC	12693	38724674
LGG	12701	32181151
LIHC	12475	38971212
LUAD	12615	30843054
PRAD	12674	34536674
STAD	12716	39856779
THCA	12655	37701765
UCES	2650	1026721

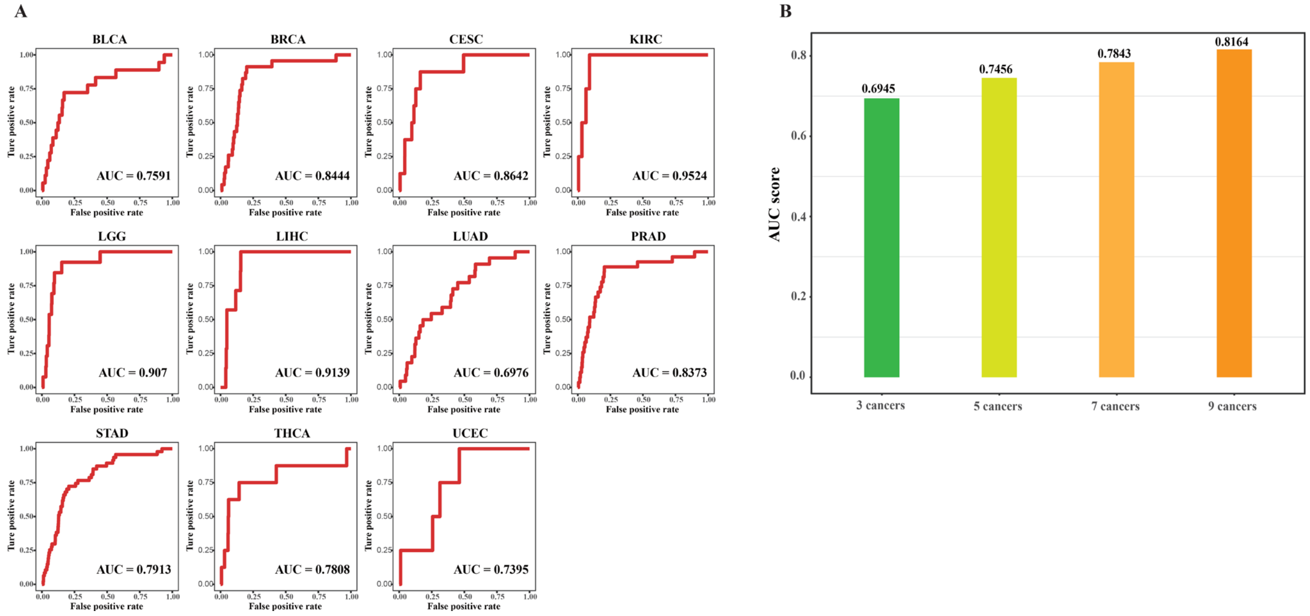

Supplementary Figure 1: Results of prediction and randomly selected cancer types. (**A**) The lncRNA prediction results calculated only using other disease information. (**B**) The prioritization results through random selection of 3, 5, 7, and 9 cancer types.

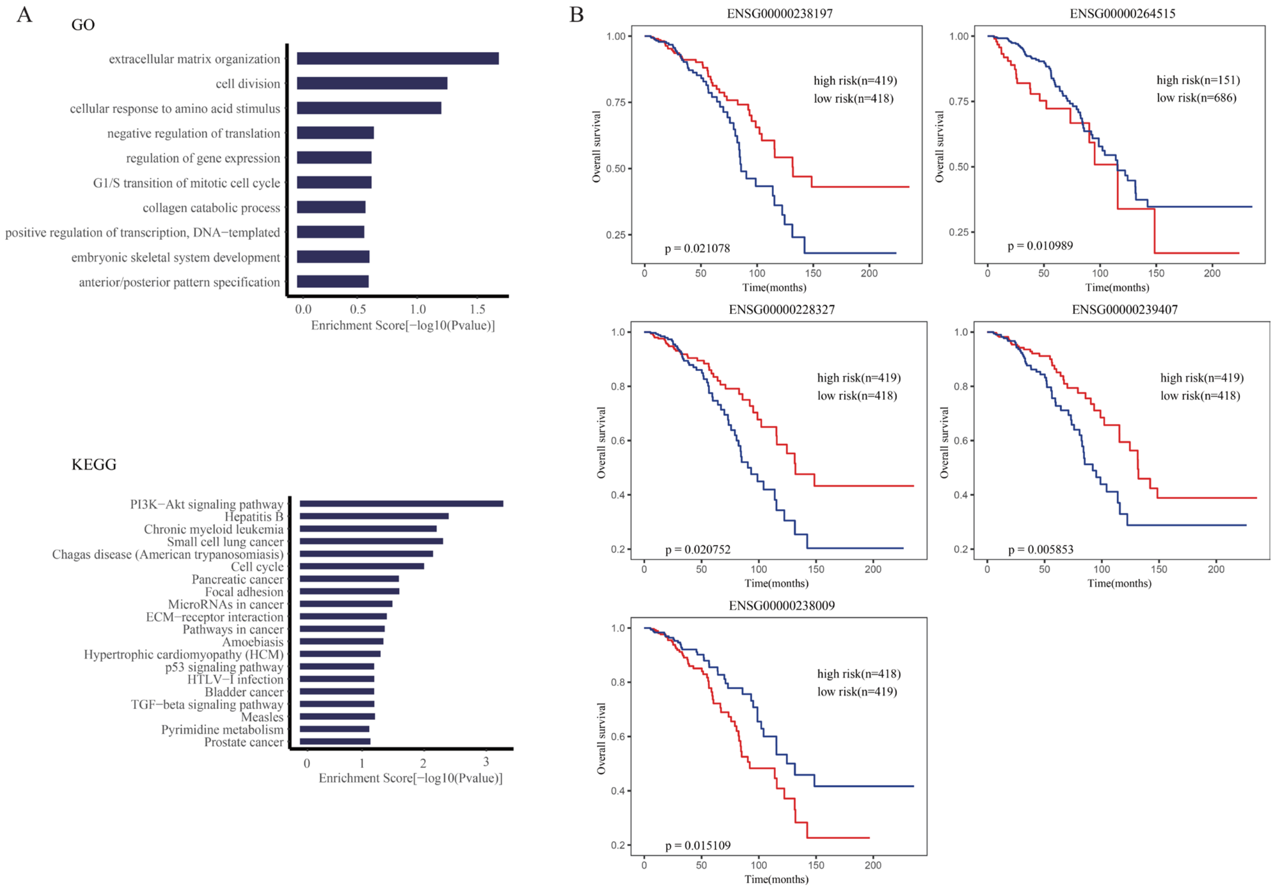

Supplementary Figure 2: The prioritization results in the case study of BRCA. (**A**) The GO and KEGG enrichment analysis results for top 10% lncRNAs of BRCA. (**B**) Survival analysis results of five candidate lncRNAs.

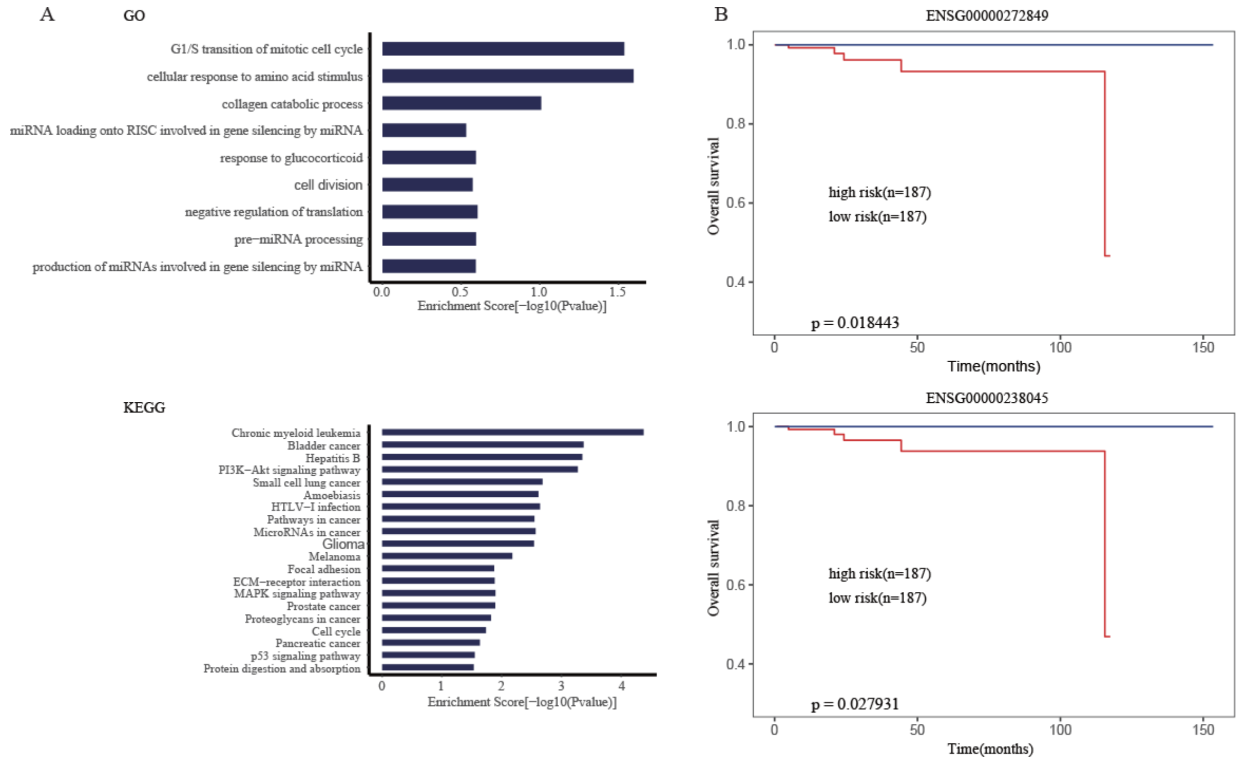

Supplementary Figure 3: The prioritization results in the case study of PRAD. (**A**) The GO and KEGG enrichment analysis results for top 10% lncRNAs of PRAD. (**B**) Survival analysis results of two candidate lncRNAs.
